# Supplementary material for: Evaluating large language models on multimodal chemistry olympiad exams
Source: Commun Chem. 2025 Dec 13;8:402. doi: 10.1038/s42004-025-01782-x (PMC12717038; doi:10.1038/s42004-025-01782-x)
Supplement: Supplementary file 1 — Description of Additional Supplementary Files [file 42004_2025_1782_MOESM1_ESM.pdf]

Description of Additional Supplementary Files:

File: Supplementary Data 1

Description: Source data of all figures presented in the paper.
